# VPA and TSA Interrupt the Interplay between mutp53 and HSP70, Leading to CHK1 and RAD51 Down-Regulation and Sensitizing Pancreatic Cancer Cells to AZD2461 PARP Inhibitor

**DOI:** 10.3390/ijms23042268

**Published:** 2022-02-18

**Authors:** Maria Anele Romeo, Maria Saveria Gilardini Montani, Rossella Benedetti, Andrea Arena, Gabriella D’Orazi, Mara Cirone

**Affiliations:** 1 Department of Experimental Medicine, Sapienza University of Rome, Viale Regina Elena 324, 00161 Rome, Italy; mariaanele.romeo@uniroma1.it (M.A.R.); mariasaveria.gilardinimontani@uniroma1.it (M.S.G.M.); rossella.benedetti@uniroma1.it (R.B.); a.arena@uniroma1.it (A.A.); 2 Laboratory Affiliated to Istituto Pasteur Italia-Fondazione Cenci Bolognetti, Viale Regina Elena 291, 00161 Rome, Italy; 3 Department of Neurosciences, Imaging and Clinical Sciences, University G. D’Annunzio, Via dei Vestini 33, 66100 Chieti, Italy; gdorazi@unich.it; 4 Department of Research and Technological Innovation, IRCCS Regina Elena National Cancer Institute, Via Elio Chianesi 53, 00128 Rome, Italy

**Keywords:** pancreatic cancer, HDACi, PARPi, mutp53, HSP70, RAD51, CHK1

## Abstract

HDAC inhibitors (HDACi) represent promising anti-cancer treatments, as the acetylation of histone and non-histone proteins is often dysregulated in cancer and contributes to cancer onset and progression. HDACi have been also reported to increase the cytotoxicity of DNA-damaging agents, such as radiation or cisplatin. In this study, we found that TSA and, even more effectively, VPA synergized with AZD2461, PARP1, 2 and 3 inhibitor (PARPi) to induce DNA damage and reduce pancreatic cancer cell survival. At a molecular level, VPA and TSA down-regulated CHK1 and RAD51, which is correlated with the interruption of the cross-talk between mutp53 and HSP70. Moreover, VPA and to a lesser extent TSA reactivated wtp53 in these cells, which contributed to CHK1 and RAD51 reduction. These findings suggest that the combination of HDACi and PARPi might improve the treatment of pancreatic cancer, which remains one of the most aggressive and therapy-resistant cancers.

## 1. Introduction

Pancreatic cancer is characterized by an aggressive behavior and a poor response to chemotherapies to which contribute the activating mutations of proto-oncogenes and/or the inactivating mutations of tumor-suppressor genes that frequently occur in this cancer [1]. Among the latter, there are mutations of the *p53* gene that often affect its DNA-binding domain. As a consequence of these so-called “hot-spot mutations”, p53 not only loses its genome guardian activity, but contributes to oncogenesis, i.e., by engaging cross-talks with pro-oncogenic molecular pathways [2]. However, in some cases, the presence of mutp53 may turn out to be an Achilles’s heel, as, for example, the R273H p53 mutation has been reported to render breast cancer cells more susceptible to treatment with poly (ADP-ribose) polymerase (PARP) inhibitors [3]. However, regarding this topic, there are contradictory studies, including our own performed on colon cancer, which suggests that mutp53 could counteract the cytotoxicity of PARP inhibitors rather than increase it [4] or not influence the PARP inhibitor treatment at all [5]. It seems that, depending on the specific cellular contexts, mutp53 may differently influence the response of cancer cells to PARP inhibitor treatment.

The PARP family proteins play a key role in DNA damage repair, mainly in the repair of single-strand breaks, which can progress to double-strand breaks if not promptly repaired. PARPs also contribute to non-homologous end joining (NHEJ) and, to lesser extent, to homologous repair (HR) [6], the two main pathways mediating DNA double-strand break repair. Cancer cells carrying mutations in DNA repair genes, for example, the BReast CAncer gene 1 (BRAC-1), a protein that belongs to the HR pathway, are known to become particularly susceptible to treatment with PARP inhibitors [7]. Based on the property of this molecule, the concept of ‘BRCAness’ has been introduced to indicate alterations in DNA repair that increase the susceptibility of sporadic cancers to PARP1 inhibitors [8].

Histone deacetylase inhibitors (HDACi) are a heterogeneous class of drugs that mediate epigenetic changes, affecting the acetylation of histones and of several other non-histone proteins [9]. HDACi, particularly those able to inhibit all HDACs, the so-called “pan HDACi”, are reported to reduce the expression level of several DNA damage repair molecules [8,10]. They may down-regulate molecules, such as BRCA-1, Checkpoint kinase 1 (CHK1) and RAD51, at a transcriptional level [11] or by increasing the acetylation of HSP90 that impairs its chaperoning function and consequently decreases the stability of DNA damage response (DDR) proteins [12]. Such effect could also occur through changes in the acetylation of HDAC6, a positive regulator of heat shock protein 90 (HSP90), at least for those HDACi that target HDAC6. Interestingly, by interfering with the acetylation of HDAC6/HSP90 complex, HDACi have been reported to reduce also the stability of mutp53 [13] and, in some cases, to reactivate wtp53 in mutp53-carrying cancer cells [14]. However, how mutp53 or wtp53 influence the expression of DDR proteins in cancer cells undergoing HDACi treatment remains to be elucidated. HDAC6 can also control the expression of HSPs, including HSP70 [15], a chaperone that contributes to the stability of mutp53 [16], particularly through its cooperation with HSP90 [17].

In previous studies, we have shown that HDACi efficiently reduced pancreatic cancer cell survival [18,19] and studies from other groups have reported that HDACi may render this cancer type more susceptible to the cytotoxic effect of DNA damaging agents, such as gemcitabine [20]. However, whether HDACi could sensitize pancreatic cancer to PARP inhibitors remains to be explored. In this paper, to investigate this aspect, we use pan-HDACi Trichostatin A (TSA) and class I/IIa HDAC inhibitor valproic acid (VPA) in combination with AZD2461, a PARP1, 2 and 3 inhibitor. At a molecular level, we evaluate the impact of these HDACi treatments on the expression of RAD51, a molecule strongly involved in HR repair, and of CHK1, responsible for cell cycle arrest, which allow the execution of such repair. The expression of these DDR molecules was correlated with that of mutp53, HSP90, and HSP70, and the reactivation of wtp53, which is known to regulate DDR [21,22].

## 2. Results

### 2.1. VPA and TSA Increase the Cytotoxic Effect and DNA Damage Induced by AZD2461 PARP Inhibitor

**Figure 1:**
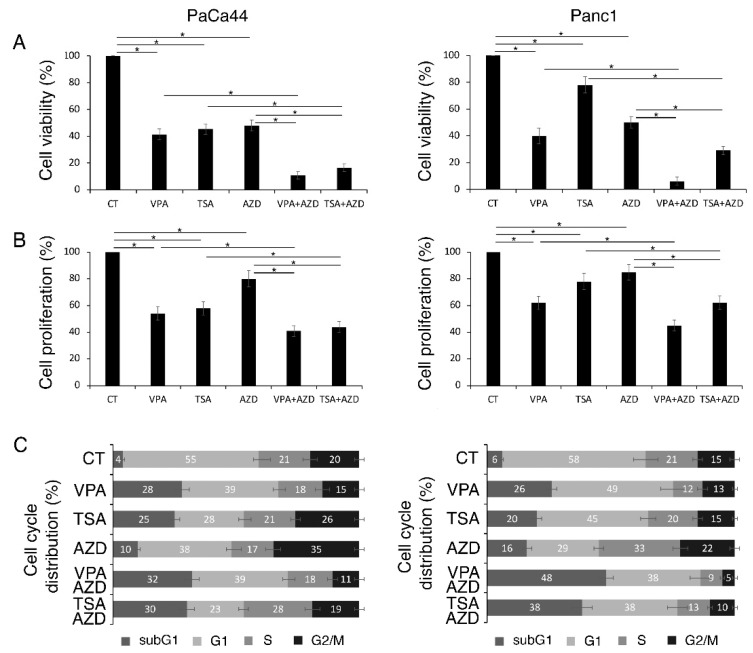
VPA and TSA increase the cytotoxic effect of AZD2461 PARP1 inhibitor. PaCa44 and Panc1 cells were pre-treated with VPA (10 mM) and TSA (500 nM) for 1 h and then treated or not with AZD (10 μM). (**A**) Cell viability was evaluated by trypan blue assay after 36 h of treatments, * *p*-value < 0.05. (**B**) Cell proliferation was evaluated by MTT assay after 36 h of treatments; * *p*-value < 0.05. (**C**) Cell cycle analysis was evaluated by FACS analysis after 36 h of treatments, following staining with PI. One representative experiment out of three is shown. The bars represent the mean of the percentage of cells in each phase of cell cycle (subG1, G1, S and G2) plus S.D. of three experiments.

HDACi have been reported to induce DNA damage that, differently from normal cells, cancer cells are not able to repair [23]. In this study, we investigate whether the cytotoxic effect previously shown to be induced by VPA and TSA against pancreatic cancer cells [18] could further increase in combination with AZD2461 PARP inhibitor (10 μM) and could correlate with the induction of a stronger DNA damage. As indicated by trypan blue (Figure 1A) and tetrazolium salt (3-(4,5-dimethylthiazol-2-yl)-2,5-diphenyltetrazolium bromide (MTT) assay (Figure 1B), the combination of AZD2461 with TSA and even more with VPA synergized in reducing PaCa44 and Panc1 cell survival. Such effect was accompanied by an increase in subG1 events in both cell lines, suggesting the occurrence of a stronger pro-apoptotic effect (Figure 1C). We then explored the impact of the HDACi/AZD2461 combination on DNA damage.

**Figure 2:**
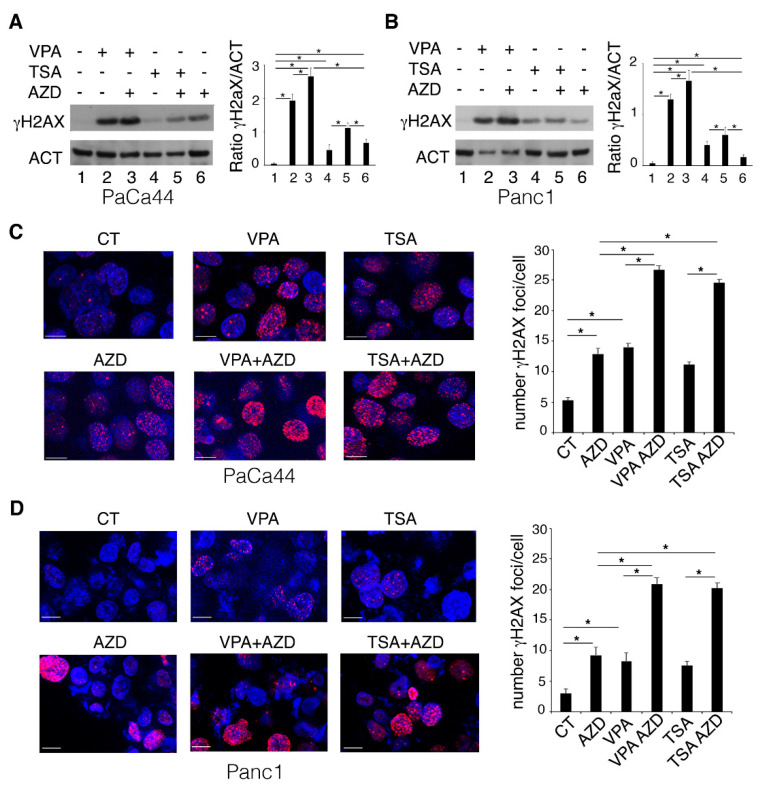
VPA and TSA increase the DNA damage induced by AZD2461 PARP1 inhibitor. PaCa44 and Panc1 cells were pre-treated with VPA (10 mM) and TSA (500 nM) for 1 h and then treated or not with AZD (10 μM). (**A**,**B**) phosphorylation of H2AX was evaluated by Western blot analysis after 36 h of treatments; actin was used by loading control. The histograms represent the mean plus S.D. of the densitometric analysis of the ratio between the protein and the appropriate control. * *p*-value < 0.05. (**C**,**D**) γH2AX foci (red) were assessed by IFA. DAPI (blue) was used for nuclear staining. Images were captured under ApoTome microscope at ×40 magnification. Bars = 10 μm. The number of foci/cells, calculated as mean plus S.D. from three independent experiments, are represented by the histograms shown on the right side of the figures C (PaCa44) and D (Panc1). * *p*-value < 0.05.

With this aim, the expression level of phosphorylated H2A histone family member X (γH2AX) and the number of γH2AX-positive foci were evaluated in pancreatic cancer cells treated by HDACi alone or combination with AZD2461. As shown in Figure 2, both γH2AX expression (Figure 2A,B) and the number of γH2AX-positive foci (Figure 2C,D) were induced by VPA and TSA and further increased following exposure to the HDACi/AZD2461 combination, suggesting the occurrence of stronger DNA damage.

### 2.2. VPA and TSA Down-Regulate RAD51 and CHK1 and Reduce the Formation of RAD51-Positive Foci in Pancreatic Cancer Cells

**Figure 3:**
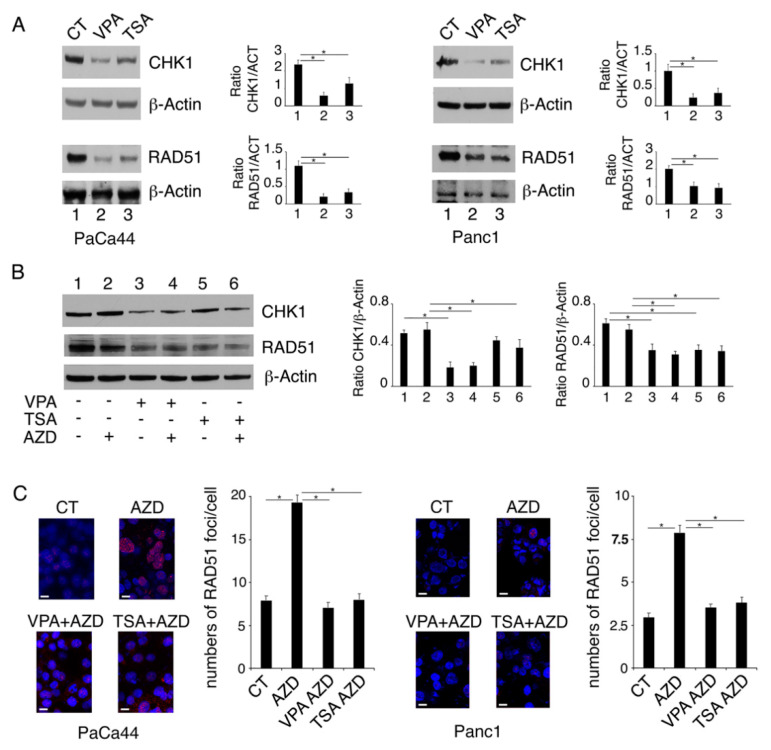
VPA and TSA downregulate RAD51 and CHK1 in pancreatic cancer cells. PaCa44 and Panc1 cells were treated with VPA (10 mM) and TSA (500 nM) for 36 h (**A**); the protein expression of CHK1 and RAD51 was evaluated by Western blot analysis. Untreated cells were used as control (CT). Actin was used as loading control. The histograms represent the mean plus S.D. of the densitometric analysis of the ratio between the protein and the appropriate control. * *p*-value < 0.05. PaCa44 cells were pre-treated with VPA (10 mM) and TSA (500 nM) for 1 h and then treated or not with AZD (10 μM) and (**B**) CHK1 and RAD51 expression was evaluated in PaCa44 by Western blot analysis. Actin was used as loading control. The histograms represent the mean plus S.D. of the densitometric analysis of the ratio between the protein and the appropriate control. * *p*-value < 0.05. (**C**) RAD51 foci (red) were evaluated by IFA in PaCa44 and Panc1. DAPI (blue) was used for nuclear staining. Images were captured under ApoTome microscope at ×40 magnification. Bars = 10 μm. The histograms represent the mean plus S.D. of the number of foci/cells from three independent experiments. * *p*-value < 0.05.

We then investigated whether the DNA damage induced by VPA and TSA could depend on an altered expression of CHK1, a kinase responsible for cell cycle arrest, and of RAD51, an enzyme essential for DNA double-strand break repair, as these molecules were previously reported to be affected by the pan-HDACi panobinostat [11]. We found that both CHK1 and RAD51 were down-regulated by VPA and to a lesser extent by TSA in both PaCa44 and Panc1 cells (Figure 3A). AZD2461 treatment slightly influenced the expression level of these DDR molecules either alone or in combination with TSA or VPA (Figure 3B). Accordingly, a reduced number of RAD51-positive foci was formed following the treatment with the HDACi/AZD2461 combination in comparison to AZD2461 alone (Figure 3C). This is in agreement with the fact that PARPs mainly contribute to the DNA single-strand break repair and the NHEJ double-strand break repair pathway. As VPA or TSA reduce CHK1 and RAD51, which is an HR repair molecule, their combination with AZD2461 could underlie the stronger DNA damage and the increased cytotoxicity observed against pancreatic cancer cells.

### 2.3. Proteasomal Degradation Is Involved in the Downregulation of CHK1 and RAD51

**Figure 4:**
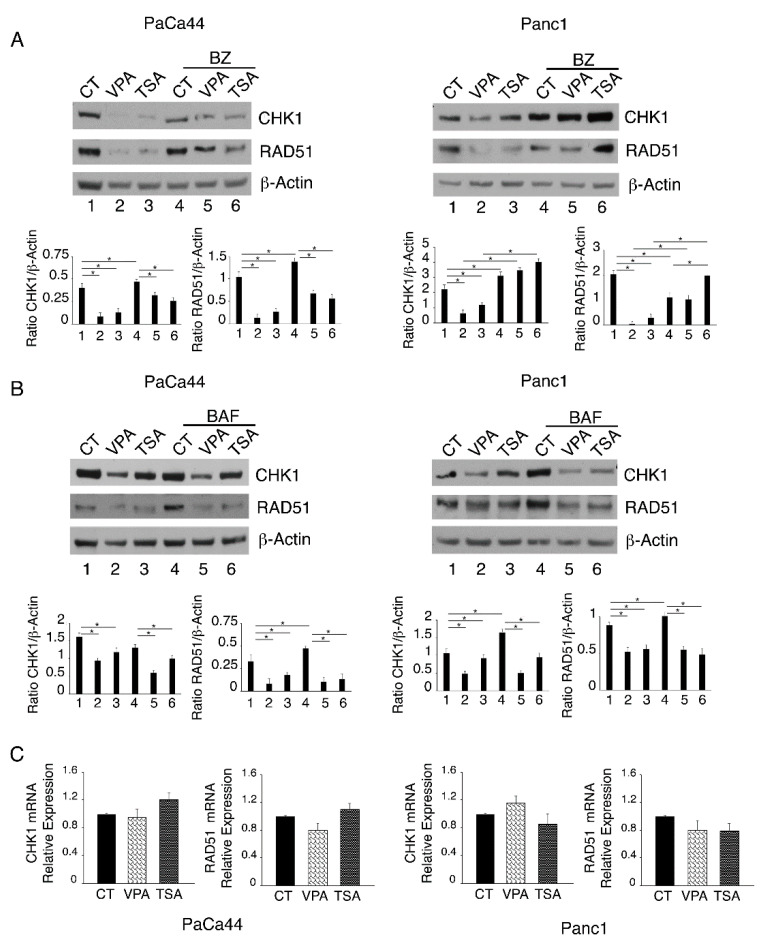
Proteasomal degradation is involved in the downregulation of RAD51 and CHK1. To evaluate proteosomal degradation, (**A**) CHK1 and RAD51 expression was evaluated by Western blot analysis in PaCa44 and Panc1 cells treated with VPA (10 mM) and TSA (500 nM) for 33 h and then with bortezomib (BZ) for the last 3 h of treatments. The histograms represent the mean plus S.D. of the densitometric analysis of the ratio between the protein and the appropriate control. * *p*-value < 0.05. To evaluate autophagy degradation, (**B**) CHK1 and RAD51 expression was evaluated by Western blot analysis in PaCa44 and Panc1 cells treated with VPA (10 mM) and TSA (500 nM) for 33 h and then with bafilomycin (BAF) for the last 3 h of treatments. Actin was used as loading control. The histograms represent the mean plus S.D. of the densitometric analysis of the ratio between the protein and the appropriate control. * *p*-value < 0.05. (**C**) mRNA expression of CHK1 and RAD51 was evaluated in PaCa44 and Panc1 cells treated with VPA (10 mM) and TSA (500 nM) for 36 h. One representative experiment out of three is shown. Data are represented as the mean relative to the control plus S.D.

We then investigated whether CHK1 and RAD51 down-regulation could be due to an increased protein degradation, via proteasomal or lysosomal routes. As shown in Figure 4A, the proteasome inhibitor bortezomib (BZ) counteracted the reduction in CHK1 and RAD51, while the bafilomycin (BAF), which interferes with lysosome function, slightly affected their expression level (Figure 4B). This suggests that these DDR molecules were mainly degraded via proteasome following VPA and TSA treatment. We then analyzed CHK1 and RAD51 mRNA by performing a qRT-PCR and found that both HDACi slightly influenced CHK1 and RAD51 mRNA (Figure 4C), suggesting that the transcriptional regulation of these proteins played a minor role in this setting. 

### 2.4. Both mutp53 Reduction and wtp53 Reactivation by VPA and TSA Contribute to the Down-Regulation of RAD51 and CHK1

**Figure 5:**
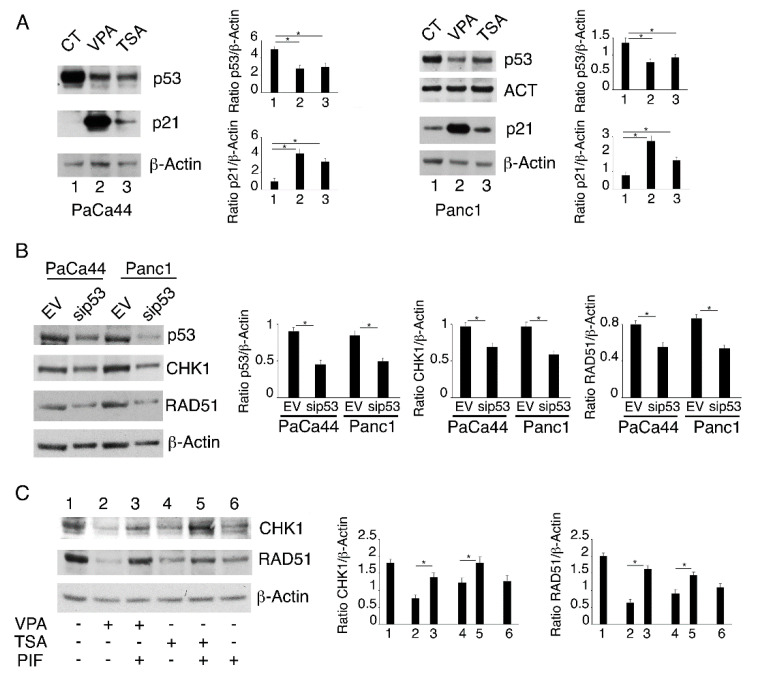
VPA and TSA cause mutp53 reduction and wtp53 reactivation that contribute to the down-regulation of RAD51 and CHK1. (**A**) p53 and p21 expression was evaluated by Western blot analysis in PaCa44 and Panc1 cells treated with VPA (10 mM) and TSA (500 nM) for 36 h. Actin was used by loading control. The histograms represent the mean plus S.D. of the densitometric analysis of the ratio between the protein and the appropriate control. * *p*-value < 0.05. (**B**) The expression of p53, CHK1 and RAD51 was evaluated in PaCa44 and Panc1 knocked-down for p53. Empty-vector transfected cells were used as control (EV). Actin was used by loading control. The histograms represent the mean plus S.D. of the densitometric analysis of the ratio between the protein and the appropriate control. * *p*-value < 0.05. PaCa44 and Panc1 cells were pre-treated with pifithrin-a (PIF) (20µM) for 1 h and then treated with VPA (10 mM) and TSA (500 nM). (**C**) The expression of CHK1 and RAD51 was evaluated in treated PaCa44 cells after 36 h. Actin was used by loading control. The histograms represent the mean plus S.D. of the densitometric analysis of the ratio between the protein and the appropriate control. * *p*-value < 0.05. 2.5 mutp53/HSP70 cross-talk down-regulates CHK1 and RAD51.

We have previously shown that VPA and TSA reduced the expression level of mutp53 in PaCa44 and Panc1 cell lines [18]. In this paper, we confirmed this finding and showed that such treatments induced a concomitant reactivation of wtp53, particularly following VPA treatment, which strongly up-regulated the expression level of p21, the p53 target (Figure 5A). To evaluate whether the mutp53 reduction could play a role in the DDR molecule down-regulation mediated by VPA or TSA, we knocked down mutp53 by specific siRNA and found that both CHK1 and RAD51 expression levels were reduced in p53-silenced cells (Figure 5B). This suggests that mutp53 was sustaining the expression level of these proteins. The possible involvement of wtp53 reactivation in the down-regulation of CHK1 and RAD51 was also explored. For this purpose, the cells were pre-treated with the wtp53 inhibitor pifithrin before exposure to VPA or TSA and, as shown in Figure 5C, pifithrin counteracted the down-regulation of CHK1 and RAD51 induced by VPA or TSA. This suggests that, in addition to mutp53 reduction, wtp53 reactivation contributed to the down-regulation of CHK1 and RAD51 observed in the HDACi-treated pancreatic cancer cells. 

**Figure 6:**
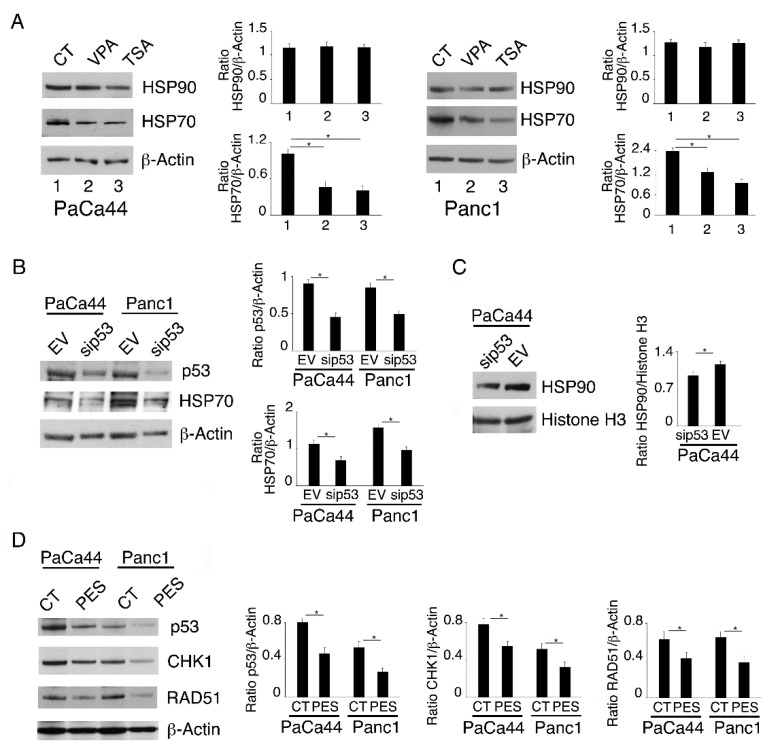
Interplay between HSP70 and mutp53 down-regulates CHK1 and RAD51 in pancreatic cancer cells treated by VPA or TSA. PaCa44 and Panc1 cells were treated with VPA (10 mM) and TSA (500 nM) for 36 h. (**A**) HSP90 and HSP70 expression was evaluated by Western blot analysis. Untreated cells were used as control (CT). Actin was used as loading control. The histograms represent the mean plus S.D. of the densitometric analysis of the ratio between the protein and the appropriate control. * *p*-value < 0.05. (**B**) p53 and HSP70 expression was evaluated in PaCa44 and Panc1 cells knocked down for p53. Empty-vector transfected cells were used as control (EV). Actin was used as loading control. The histograms represent the mean plus S.D. of the densitometric analysis of the ratio between the protein and the appropriate control. * *p*-value < 0.05. (**C**) The expression of HSP90 was evaluated in PaCa44 cells knocked down for p53. Empty-vector transfected cells were used as control (EV) and actin was used as loading control. The histograms represent the mean plus S.D. of the densitometric analysis of the ratio between the protein and the appropriate control. * *p*-value < 0.05. To evaluate the role of HSP70, PaCa44 and Panc1 cells were treated with 2-phenylethynesulfonamide (PES) (HSP-70 inhibitor) (10 μM) for 24 h. (**D**) p53, CHK1 and RAD51 expression was evaluated by Western blot analysis. Untreated cells were used as control (CT). Actin was used as loading control. The histograms represent the mean plus S.D. of the densitometric analysis of the ratio between the protein and the appropriate control. * *p*-value < 0.05.

mutp53 is known to be an HSP90 client [24], as the proper expression/function of HSP90 is required to maintain mutp53 stability. In this paper, we investigated whether mutp53 downregulation mediated by VPA or TSA could correlate with a down-regulation of HSP90. As shown in Figure 5A, the expression level of this chaperone was not affected, while differently from this HSP, HSP70 was down-regulated by both HDACi (Figure 6A). The down-regulation of HSP70 can play a role in the reduction in mutp53, as HSP70 cooperates with HSP90 in the stabilization of its numerous client proteins [17]. Interestingly, the silencing of mutp53 reduced HSP70 (Figure 6B), suggesting a positive regulatory circuit between the two proteins. Moreover, we found that the knockdown of mutp53 also partially down-regulated HSP90 (Figure 6C), as previously reported [24]. To demonstrate the importance of this chaperone in maintaining the stability of mutp53, we used an HSP70 inhibitor, 2-phenylethynesulfonamide (PES), and found that such treatment reduced mutp53 expression levels (Figure 6D). Interestingly, CHK1 and RAD51 were also down-regulated by PES treatment (Figure 6D). All together these findings suggest that the interplay between HSP70 and mutp53 strongly contributes to sustain the expression of these DDR molecules.

## 3. Discussion

Combination therapies may represent a promising option to treat cancers, especially those highly resistant to anti-cancer treatments, such as pancreatic cancer. HDACi may be good candidate to be used in combination therapies given that, by affecting the acetylation of histones and non-histone molecules, they influence a variety of biological processes, from gene transcription to DNA damage repair, signal transduction, protein folding and autophagy [25,26]. The dysregulation of deacetylases frequently occurs in cancers and contributes to cancer onset, progression and response to therapies [10]. The targeting of HDACs has been demonstrated to be an effective anticancer approach as single agents, against pancreatic [18,27] as well as other solid [13] or hematologic cancer types [11,28]. Based on their capacity to interfere with DNA repair [23], HDACi have been introduced also in combination anti-cancer therapies together with DNA damaging agents, such as cisplatin, doxorubicin [29,30] or ionizing radiation [31]. Moreover, HDACi have been shown to increase the susceptibility to PARP inhibitors against cancers, such as triple negative breast cancer [8,12] or prostatic cancer [32,33].

In this study, we found that PARP inhibitors and HDCi could be a promising approach also against pancreatic cancer. Indeed, VPA, that selectively inhibits class I and IIa HDACs and to a lesser extent the pan HDACi TSA, increased the cytotoxicity of AZD2461, PARP1, 2 and 3 inhibitor. Such effect correlated with the induction of a stronger DNA damage in cells treated by the HDACi/AZD2461 combination, due to the down-regulation of CHK1 and RAD51 by VPA and TSA. The finding that VPA was more effective than TSA suggests that HDAC I and IIa might play the major role in sustaining the expression level of these DDR molecules. The stronger cytotoxicity induced by of the HDACi and PARPi combination may depend on the fact that one of the mechanisms underlying the resistance of cancers to PARP inhibitors treatment is the activation of HR DNA repair pathway [34], that we show in this paper to be impaired by VPA and TSA. Another interesting aspect of this study is that the down-regulation of CHK1 and RAD51 correlated with the interruption of the interplay between mutp53 and HSP70 by these HDACi. Although the expression of HSP90, one of the main HSP stabilizing mutp53 [26], was not altered, it must be considered that HSP70 strongly cooperates with HSP90 to chaperone its client proteins [17], including those belonging to DDR pathways [35]. The importance of HSP70 was demonstrated in this study by the use of PES, a HSP70 inhibitor, which efficiently reduced the expression level of mutp53 as well as that of CHK1 and RAD51. In previous studies, HDACi have been reported to reduce the expression of DDR molecules at a transcriptional level [11], a mechanism that played a minor role in our setting. Indeed, we found that an increased degradation via proteasome was responsible for CHK1 and RAD51 down-regulation following the VPA and TSA treatment of pancreatic cancer cells.

According to previous studies [13], a dysregulated acetylation of HDAC6, a partner of HSP90, could contribute to the reduction in these DDR proteins by TSA, as, differently from VPA, it is able to target HDAC6. Interestingly, VPA and to a lesser extent TSA reactivated wtp53 in pancreatic cancer cells carrying it in a mutant state, and such effect further contributed to the reduction in CHK1 and RAD51. In agreement with these results, the inhibition of HR molecules, such as BRCA1 and RAD51, by wtp53 activation has been demonstrated in previous studies in other cancer cell types [4,21,22].

## 4. Materials and Methods

### 4.1. Cell Cultures and Treatments

PaCa44 cell line (pancreatic ductal adenocarcinoma) (kindly provided by Dr. M. von Bulow University of Mainz, Mainz, Germany) with mutp53 (C176S) and Panc1 cell line (pancreatic ductal adenocarcinoma) (provided by ATCC, USA) with mutp53 (R273H) were maintained in RPMI 1640 supplemented with 10% fetal bovine serum (FBS) (Corning, USA), L-glutamine, streptomycin (100 μg/mL) (Aurogene, Rome, Italy), and penicillin (100 U/mL) (Aurogene, Rome, Italy) (complete medium, CM) in 5% CO_2_ at 37 °C. Cells were always detached using Trypsin-EDTA solution (Aurogene, Rome, Italy). Cells were plated in 6-well plates at a density of 2 × 105 cells/well in 2 mL of CM and, the day after, treated with valproic acid (VPA) (10 mM) (Sigma Aldrich, Burlington, MA, USA) and trichostatin A (TSA) (500 nM) (Sigma Aldrich, Burlington, MA, USA) for 36 h. In some experiments, cells, plated as described, were pre-treated with VPA (10 mM) and TSA (500 nM) for 1 h and then treated with AZD2461 (10 μM) (Sigma Aldrich, Burlington, MA, USA). In some experiments, cells were treated with VPA (10 mM) and TSA (500 nM) for 33 h and in the last 3 h of culture were treated with bortezomib (BZ) (10 nM) (Sigma Aldrich, Burlington, MA, USA) or bafilomycin (BAF) (20 nM) (Sigma Aldrich, Burlington, MA, USA). To evaluate the role of p53, cells were pre-treated with pifithrin-a (PIF) (20µM) (Sigma Aldrich, Burlington, MA, USA) for 1 h and then treated with VPA (10 mM) and TSA (500 nM) (Sigma Aldrich, Burlington, MA, USA). The results were evaluated after 36 h of treatments. To evaluate the role of HSP70, cells were plated as explained before and then treated with 2-phenylethynesulfonamide (PES) (HSP70 inhibitor) (10 μM) (Sigma Aldrich, Burlington, MA, USA) for 24 h. In all experiments, untreated cells were used as control (CT).

### 4.2. Trypan Blue Assay

After treatments with VPA, TSA and AZD2461, the cell viability was evaluated using a trypan blue exclusion assay (Sigma Aldrich, Burlington, MA, USA). Cells were counted by light microscopy using a Neubauer hemocytometer. The experiments were performed in triplicate and repeated at least three times.

### 4.3. MTT Assay

Cell proliferation was evaluated by MTT assay (Sigma Aldrich, Burlington, MA, USA). A total of 5 × 103 cells/well were plated in 96-well plates in 100 μL of complete medium. The day after, the cells were pre-treated with VPA (10 mM) and TSA (500 nM) for 1 h and then treated with AZD2461 (10 μM) (Sigma Aldrich, Burlington, MA, USA) for 72 h. Untreated cells were used as control. MTT assay was performed following manufacturer’s instruction. The plates were analyzed by Byonoy Absorbance 96 (Germany). The experiments were performed in triplicate and repeated three times.

### 4.4. p53 Silencing

PaCa44 and Panc1 cells were seeded into 6-well plates at a density of 2 × 105 cells/well and transfected, the following day, with an empty vector (p-Super) or sip53 plasmid (p-Super-p53) [36] for p53 knockdown using lipofectamine 2000 (Invitrogen, Waltham, MA, USA), according to the manufacturer’s instructions. After 48 h, the cells were recovered for further analysis.

### 4.5. Cell Cycle Analysis

For cell cycle analysis, the DNA content of untreated or treated PaCa44 and Panc1 cells, was measured by propidium iodide (PI) (Sigma Aldrich, Burlington, MA, USA) staining and FACS analysis. After 36 h of treatment, the cells were washed with cold 1× PBS and fixed in 70% ethanol on ice for at least 1 h. After centrifugation, the cell pellet was washed with cold 1× PBS, stained with 50 μg/mL PI and RNase for 15 min at 37 °C and then analyzed by FACSCalibur (BD Biosciences, Franklin Lakes, NJ, USA). Data are representative of at least three independent experiments.

### 4.6. Indirect Immunofluorescence Assay (IFA)

To evaluate γH2AX and RAD51 foci formation, PaCa44 and Panc1 cells were grown on slides, treated as explained above, washed with PBS and air dried. The cells were then incubated with 2% paraformaldehyde (Electron Microscopy Science, Hatfield, PA, USA) for 30 min and permeabilized with 0.1% Triton X-100 (Sigma Aldrich, Burlington, MA, USA) for 5 min. After 3 washes, the cells were incubated with 1% glycine, 3% BSA for a further 30 min. Then, the cells were incubated with the primary monoclonal antibody against p-H2AX (Ser 139) (1:100) (Santa Cruz Biotechnology Inc., Dallas, TX, USA, sc-517348) or RAD51 (1:100) (Santa Cruz Biotechnology Inc., Dallas, TX, USA, sc-398587) for 1 h at room temperature. Slides were then washed 3 times with PBS and the cells were further incubated with a polyclonal conjugated-Cy3 sheep anti-mouse antibody (1:2000) (Jackson ImmunoResearch, UK) for 30 min at room temperature. After 3 washes in PBS, the cells were stained with DAPI (1:5000) (SIGMA) for 1 min at room temperature. Slides were further washed in PBS, mounted with glycerol:PBS (1:1) and analyzed with an Apotome Axio Observer Z1 inverted microscope (Zeiss, Germany) equipped with an AxioCam MRM Rev.3 (Germany) at 40 magnification. Foci amount was counted by Image J software (USA).

### 4.7. Western Blot Analysis

Following transfections and treatments, the cells were washed in 1X PBS, lysed in RIPA buffer (150 mM NaCl, 1% NP-40, 50 mM Tris-HCl (pH 8), 0.5% deoxycholic acid, 0.1% SDS, protease and phosphatase inhibitors) and centrifuged at 14,000× *g* rpm for 20 min at 4 °C. The protein concentration was measured by using the Bio-Rad Protein Assay (Bio-Rad laboratories GmbH) and 15 μg of protein was subjected to electrophoresis on 4–12% NuPage Bis-Tris gels (Life Technologies, Carlsbad, CA, USA), according to the manufacturer’s instruction. The gels were transferred to nitrocellulose membranes (Bio-Rad, Hercules, CA, USA) for 2 h in tris-glycine buffer and the membranes were blocked in 1 X PBS-0.1% Tween20 solution containing 3% of BSA (SERVA) probed with specific antibodies and developed using ECL Blotting Substrate (Advansta, San Jose, CA, USA) [37].

### 4.8. Antibodies

To evaluate protein expression on Western blot membranes the following antibodies were used: mouse monoclonal anti-pH2AX (Ser 139) (1:100) (Santa Cruz Biotechnology Inc., Dallas, TX, USA, sc-517348), mouse monoclonal anti-CHK1 (1:100) (clone F-11, Santa Cruz Biotechnology Inc., Dallas, TX, USA, sc-8408), mouse monoclonal anti-RAD51 (1:100) (clone G-4, Santa Cruz Biotechnology Inc., Dallas, TX, USA, sc-398587), mouse monoclonal anti-p53 (1:100) (clone DO-1, Santa Cruz Biotechnology Inc., Dallas, TX, USA, sc-126), rabbit polyclonal anti-p21 (1:200) (clone C-19, Santa Cruz Biotechnology Inc., Dallas, TX, USA, sc-397), rabbit polyclonal anti-HSP70 (1:1000) (Proteintech, Rosemont, IL, USA, 10995-1-AP) and rabbit polyclonal anti-HSP90 (1:1000) (Proteintech, Rosemont, IL, USA, 13171-1-AP). Mouse monoclonal anti-β-actin (1:10,000) (Sigma Aldrich, Burlington, MA, USA) or rabbit polyclonal anti-Histone H3 (4499, Cell Signaling, Danvers, MA, USA) were used as loading control. The goat anti-mouse IgG-HRP (1:30,000) (Bethyl Laboratories, Montgomery, TX, USA, A90-116P) and goat anti-rabbit IgG-HRP (1:30,000) (Bethyl Laboratories, Montgomery, TX, USA, A120-101P) were used as secondary antibodies. All the primary and secondary antibodies were diluted in PBS-0.1% Tween20 solution containing 3% of BSA (SERVA).

### 4.9. RNA Isolation and Quantitative Real Time Polymerase Chain Reaction (qRT-PCR)

The total RNA from PaCa44 cells treated with VPA (10 mM) and TSA (500 nM) for 36 h was isolated with TRIzol™ Reagent (Invitrogen, Life Technologies Corporation, Carlsbad, CA, USA), according to the manufacturer’s instructions. After in vitro reverse transcription (2 μg) with the High-capacity cDNA Reverse Transcription Kit (Thermo Fisher Scientific, Waltham, MA, USA), quantitative Real-Time PCR (qRT-PCR) was performed on an Applied Biosystem Real-Time and best-coverage TaqMan gene expression assays specific for each mRNA analyzed (CHK1 and RAD51). Each amplification was performed in triplicate, and the average of three threshold cycles was used to calculate transcript abundance. Transcript quantification was expressed in arbitrary units as the ratio of the sample quantity to the mean values of control samples (untreated cells). All values were normalized to β2-microglobulin (endogenous gene control).

### 4.10. Densitometric Analysis

The quantification of proteins bands was performed by densitometric analysis using the Image J software (1.47 version, NIH, Bethesda, MD, USA).

### 4.11. Statistical Analysis

The results are represented by the mean ± standard deviation (S.D.) of at least three independent experiments and a two-tailed Student’s t-test was used to demonstrate statistical significance. Difference was considered as statistically significant when *p*-value was at least <0.05.

## 5. Conclusions

This study suggests that VPA and to a lesser extent TSA synergize with the PARP inhibitor to reduce the survival of pancreatic cancers carrying mutp53, due to a stronger DNA damage induced by this combination treatment. As underlying mechanisms, we found that the interruption of mutp53/HSP70 cross-talk and wtp53 reactivation played a role in the reduction in CHK1 and RAD51 molecule stability. Interestingly, the interconnection between these effects induced by HDACi has never highlighted before in cancer cells. The findings of this study encourage the use of HDACi, especially those inhibiting class I and IIa HDACs, in combination with PARP inhibitor in the treatment of pancreatic cancer.

## Data Availability

The datasets generated and analyzed during the current study are available from the corresponding author upon reasonable request.
